# A hundred years of rabies in Kenya and the strategy for eliminating dog-mediated rabies by 2030

**DOI:** 10.12688/aasopenres.12872.2

**Published:** 2019-02-15

**Authors:** Austine O Bitek, Eric Osoro, Peninah M Munyua, Mark Nanyingi, Yvonne Muthiani, Stella Kiambi, Mathew Muturi, Athman Mwatondo, Rees Muriithi, Sarah Cleaveland, Katie Hampson, M. Kariuki Njenga, PM Kitala, SM Thumbi

**Affiliations:** 1 Zoonotic Disease Unit, Ministry of Agriculture and Livestock, Nairobi, Kenya; 2 Zoonotic Disease Unit, Ministry of Health, Nairobi, Kenya; 3 Division of Global Health Protection, Centers for Disease Control and Prevention, Nairobi, Kenya; 4 Department of Public Health, Pharmacology and Toxicology, University of Nairobi, Nairobi, Kenya; 5 Directorate of Veterinary Services, Ministry of Agriculture, Livestock and Fisheries, Nairobi, Kenya; 6 Boyd Orr Centre for Population and Ecosystem Health, Institute for Biodiversity, Animal Health & Comparative Medicine, University of Glasgow, Glasgow, UK; 7 Center for Global Health Research, Kenya Medical Research Institute, Nairobi, Kenya; 8 Paul G. Allen School for Global Animal Health, Washington State University, Pullman, WA, USA; 9 Rabies Free Africa, Washington State University, Pullman, WA, USA

**Keywords:** rabies, elimination, Kenya, epidemiology

## Abstract

**Background**: Rabies causes an estimated 59,000 human deaths annually. In Kenya, rabies was first reported in a dog in 1912, with the first human case reported in 1928. Here we examine retrospective rabies data in Kenya for the period 1912 – 2017 and describe the spatial and temporal patterns of rabies occurrence in the country. Additionally, we detail Kenya’s strategy for the elimination of dog-mediated human rabies by 2030.

**Methods**: Data on submitted samples and confirmed cases in humans, domestic animals and wildlife were obtained from Kenya’s Directorate of Veterinary Services. These data were associated with the geographical regions where the samples originated, and temporal and spatial trends examined.

**Results**: Between 1912 and the mid 1970’s, rabies spread across Kenya gradually, with fewer than 50 cases reported per year and less than half of the 47 counties affected. Following an outbreak in the mid 1970’s, rabies spread rapidly to more than 85% of counties, with a 4 fold increase in the percent positivity of samples submitted and number of confirmed rabies cases. Since 1958, 7,584 samples from domestic animals (93%), wildlife (5%), and humans (2%) were tested. Over two-thirds of all rabies cases came from six counties, all in close proximity to veterinary diagnostic laboratories, highlighting a limitation of passive surveillance.

**Conclusions**: Compulsory annual dog vaccinations between 1950’s and the early 1970’s slowed rabies spread. The rapid spread with peak rabies cases in the 1980’s coincided with implementation of structural adjustment programs privatizing the veterinary sector leading to breakdown of rabies control programs. To eliminate human deaths from rabies by 2030, Kenya is implementing a 15-year step-wise strategy based on three pillars: a) mass dog vaccination, b) provision of post-exposure prophylaxis and public awareness and c) improved surveillance for rabies in dogs and humans with prompt responses to rabies outbreaks.

## Introduction

Every year rabies is estimated to kill around 59,000 (95% CI: 25-159,000) people globally, with the vast majority of rabies deaths occurring in rural Africa and Asia ^1, 2^. Additionally, the disease is estimated to cause over 3.7 million (95% CI: 1.6–10.4 million) disability-adjusted life years (DALYs) and 8.6 billion USD (95% CI: 2.9–21.5 billion) in economic losses annually ^1^. These human and economic losses occur despite the existence of effective anti-rabies vaccines for humans and animals and data that supports the feasibility of dog-rabies elimination ^3, 4^. In areas with high canine rabies burden, human rabies remains largely underreported owing to poor surveillance and misdiagnosis with other common diseases manifesting with nervous disorders such as cerebral malaria ^1, 3, 5– 7^. Consequently, this has led to a perceived lack of importance for human rabies, driving a cycle of neglect for this endemic disease ^4^.

In Kenya, rabies has been a public health problem since the first case was reported in a dog in the outskirts of Nairobi in 1912, and in a woman from the Lake Victoria basin in 1928 ^8^. The exact number of human deaths due to rabies in Kenya is unknown, although estimates have been made for some regions of the country and as part of the global burden of rabies estimates ^1, 9, 10^. A recent review of research on human and animal rabies in Kenya revealed 12 published manuscripts and four theses on rabies covering mainly knowledge attitudes and practices on rabies, dog ecology and demographics and bite exposures ^11^. A formal assessment of zoonotic diseases in Kenya has placed rabies among the top five priority zoonotic diseases ^12^. As a result, Kenya developed a strategic plan for the prevention and elimination of dog-mediated human rabies. The strategy, adopted for implementation in 2014, provides the country with a framework for progressive reduction and eventual elimination of human deaths from rabies by 2030 ^13^, in line with set target for the global elimination of dog-mediated rabies ^14^.

Here we review the historical data on human and animal rabies in Kenya from 1912 to 2017 and examine the patterns of rabies spread across the country, and the trends over time. Additionally, we detail the strategy adopted by Kenya towards elimination of dog-mediated human rabies by the year 2030.

## Methods

### Data collection

Rabies is a notifiable disease in Kenya, and the data are obtained through a passive surveillance system. Suspected cases of animal rabies are notified immediately to the local veterinary officers who are required to fill a standardised form with epidemiologically relevant information on the cases. The forms are sent to the Director of Veterinary Services (DVS), and the samples from the suspected rabid animals are sent to either the Central Veterinary Laboratory (CVL) located in Nairobi County or to any of six Regional Veterinary Investigation Laboratories (RVIL). The RVILs are placed across the country in Nakuru, Eldoret, Kericho, Garissa, Nyeri and Kilifi Counties. After initial testing, all samples were further submitted to one of three laboratories: the Central Veterinary Laboratory (CVL), the Regional Veterinary Investigation Laboratories (RVIL) in Kericho in Western Kenya, or RVIL in Kilifi at the Coast. These three laboratories carry out the Fluorescent Antibody Test (FAT), which is the confirmatory rabies diagnostic test recommended by the World Organisation for Animal Health (OIE) ^15^. The test results were reported against the sample’s source species and county of origin.

We obtained surveillance records on human and animal samples submitted and tested for rabies by the Central Veterinary Laboratory (CVL) in Kabete and the Regional Veterinary Investigation Laboratories (RVILs) in Kenya. Historically, human rabies diagnosis in Kenya has been conducted at the veterinary laboratories as public health laboratories have lacked diagnostic capacity for rabies. We obtained records for the years 1912 – 2017 from the Kenya Directorate of Veterinary Services and extracted data on the number of samples submitted for rabies testing, dates of sample submission, animal species and humans, administrative units (counties/districts) where the samples came from, and test results. As the administrative units in Kenya have changed over time, each unit reported in the raw data was matched to a current county. The analyses were conducted per existing counties to allow for consistency in reporting. Similar data were extracted from a published book containing historical data recorded by the Directorate of Veterinary Services for the years 1912 and 1981 ^8^. These data were reviewed, cleaned and merged to provide a database with complete records on rabies in Kenya for the years 1912 – 2017. Periods where data were missing have been highlighted in the relevant results. Retrospective data analyzed in this study were from routine national surveillance for rabies. Demographic and personal identifiable data were not collected together with the samples and individual consent was not obtained. The laboratory results of analysis of human samples were analysed as monthly aggregates.

For the period prior 1958, data on the number of samples submitted for testing and the species of animals they were obtained from were not available. Only aggregate numbers of positive cases of rabies per year per county were available for analyses.

## Data analysis

We computed the proportions of human and animal samples submitted that tested positive for rabies by year, number of cases by species and administrative counties and examined spatial and temporal trends in rabies occurrence. We determined the proportion of samples submitted that were positive for rabies by year and for each county. Linear mixed-effect models with year as the fixed effect, and the county as a random effect (to account for proximity to testing laboratories) were used to test if the proportion of submitted samples that were positive for rabies changed over time. The analysis was carried out using R platform (version 3.4.0) for statistical computing ^16^.

### Strategic plan for the elimination of human rabies in Kenya

This manuscript discusses the strategies put in place for the elimination of human rabies in Kenya by the year 2030. The strategic plan was developed through an elaborate consultative process that involved government departments in health and livestock, research and academic institutions, non-governmental organizations and international partners in Kenya working on public and animal health. The detailed strategy is available online ^13^.

## Results

Between 1958 and 2017, 7,584 samples from suspect human and animals rabies cases were submitted for laboratory testing. Samples from domestic animals (cattle, dogs, sheep, goats, pigs and equine) accounted for 93% (7,013/7,584), wildlife (jackal, fox, mongoose, lions, squirrels, bats, and civet) for 5% (407/7,584) and those from humans for 2% (164/7,584) of the total samples ( Table 1).

**Table 1.** Number of samples submitted, tested for rabies and percent positivity by species, Kenya 1958 – 2017.

Species	Number of samples submitted	Number positive	% Positive
Domestic			
Dogs	4527	2796	62
Cattle	1461	1192	82
Cats	479	198	41
Goats/Sheep	361	280	78
Equine	167	113	68
Pigs	12	7	58
Camel	6	1	17
Sub-total	7013	4587	65
Wildlife			
Jackal	89	56	63
Fox	26	17	65
Honey badger	39	23	59
Hyena	15	7	47
Civet	9	3	33
Mongoose	61	38	62
Other wildlife	168	12	7
Sub-total	407	156	38
Human	164	120	73
**Total**	**7584**	**4863**	**64**

The most frequently submitted samples from domestic animals for rabies diagnosis were from dogs, comprising nearly two-thirds (4527/7,013) while those from domestic ruminants (cattle, sheep and goats) made up 26% of the samples (1822/7,013). Among samples obtained from domestic animals the overall percent positivity was 65%, while the percent positivity from wildlife was 38% and from humans was 73%. Samples from cattle, goats, sheep, and horses returned a higher percent positivity compared to those from dogs and cats ( Table 1). We could not find records for the number of samples submitted and the species they we prior to 1958.

## Temporal distribution of rabies cases by species, 1958 – 2017

**Figure 1.**
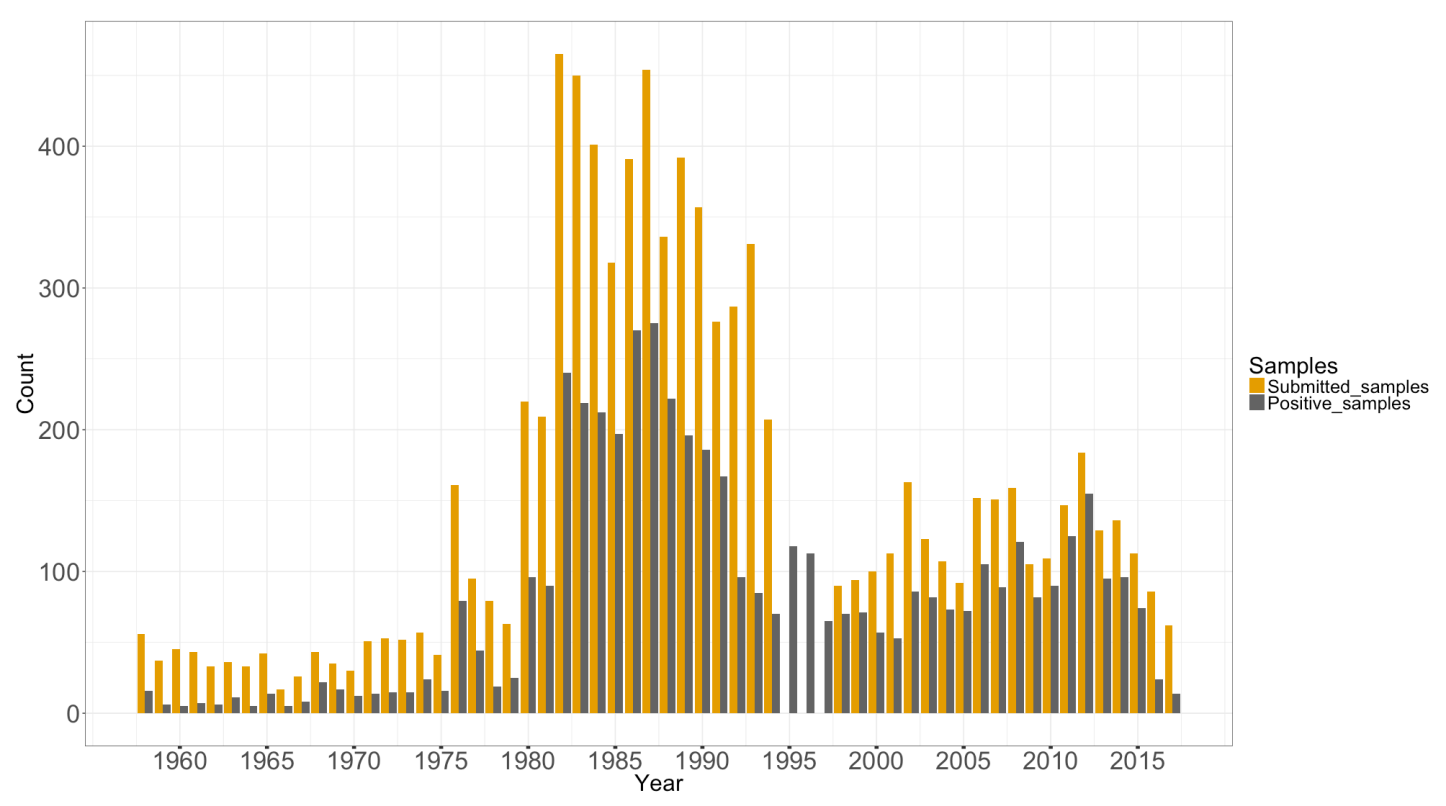
Trends in total submitted human and animal samples and confirmed rabies cases in Kenya from 1958 until 2017.

**Figure 2.**
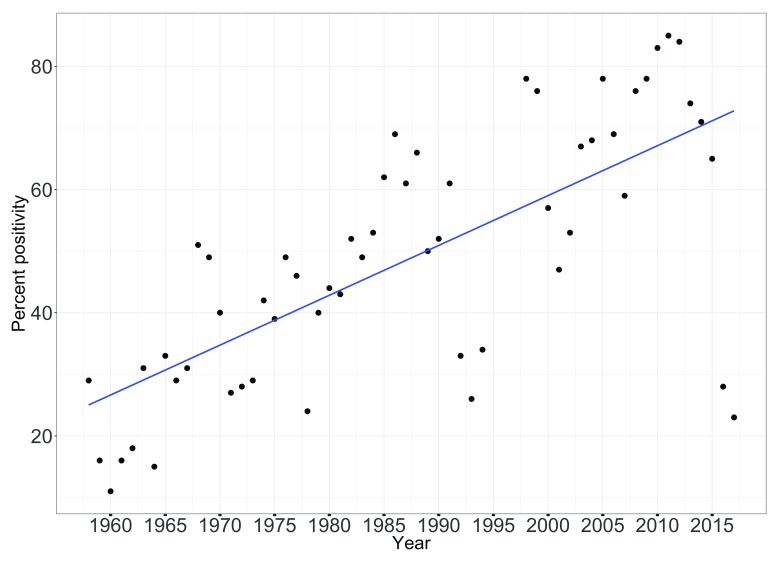
Figure showing the proportion of human and animal samples (%) submitted for rabies testing that were positive for each year 1958–2017. The proportion has steadily increased over time as shown by the regression line (blue). No records of samples submitted were available for the years 1995, 1996 or 1997.

**Figure 3.**
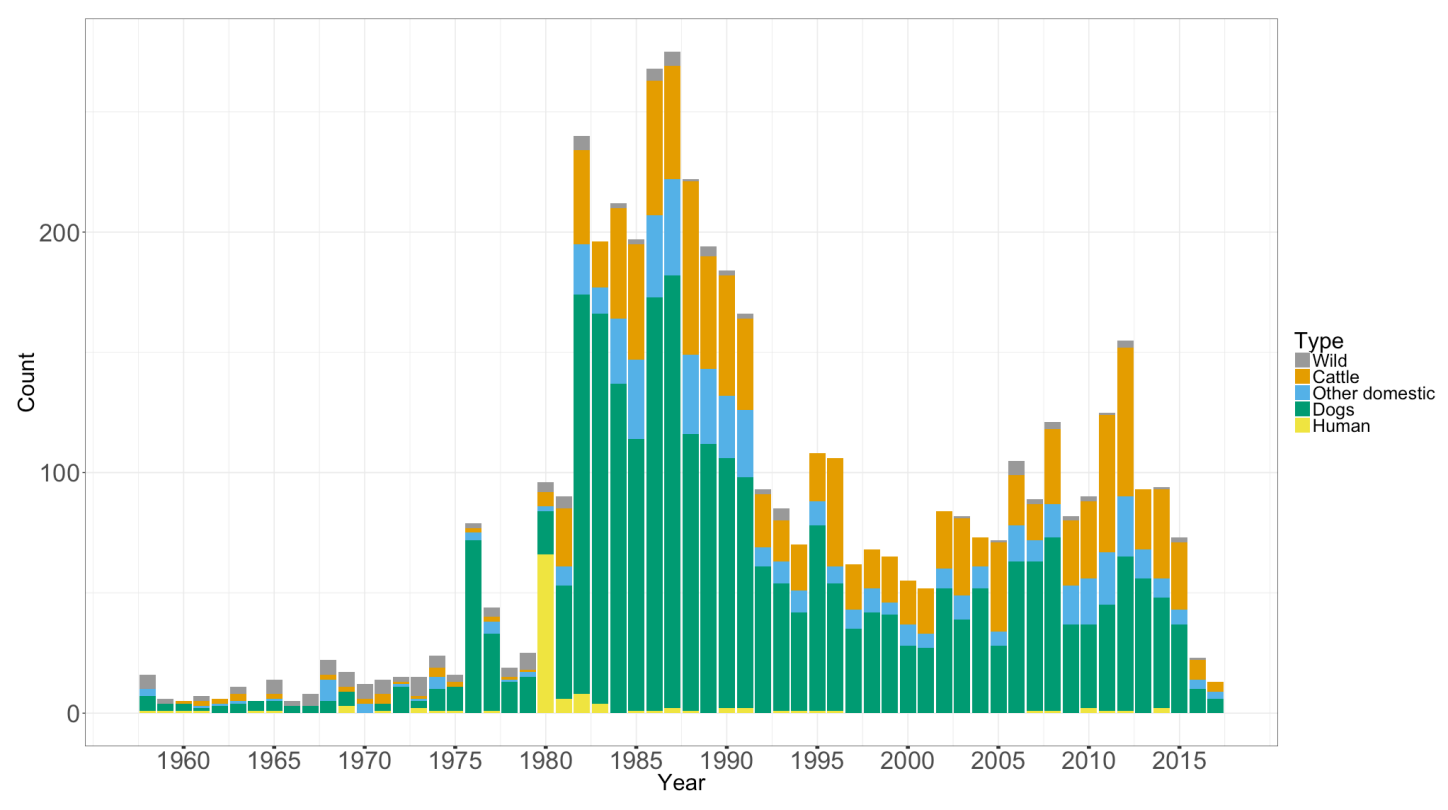
Trends of confirmed rabies cases by species for the period 1958 to 2017.

Analysis of the number of human and animal samples submitted and confirmed for rabies shows three periods with distinct patterns of rabies occurrence. First is the period from 1958 to the early 1970s where a relatively low number of cases were reported (<50 cases/year). The second is the period covering the 1980’s and the early 1990’s where more than >200cases were reported per year. The third period covers the mid 1990s to 2017 with approximately 100 confirmed cases reported per year ( Figure 1). Analyses of the percent positivity data show a general increase over time in the proportion of samples submitted that were positive for rabies ( Figure 2). The model estimates were 0.38 (95% CI 0.17, 0.59) increase in percent positivity per year. Over time, most positive cases were consistently confirmed in domestic animals, with the majority being domestic dogs ( Figure 3).

## Spatial distribution of rabies in Kenya, 1912 – 2017

**Figure 4.**
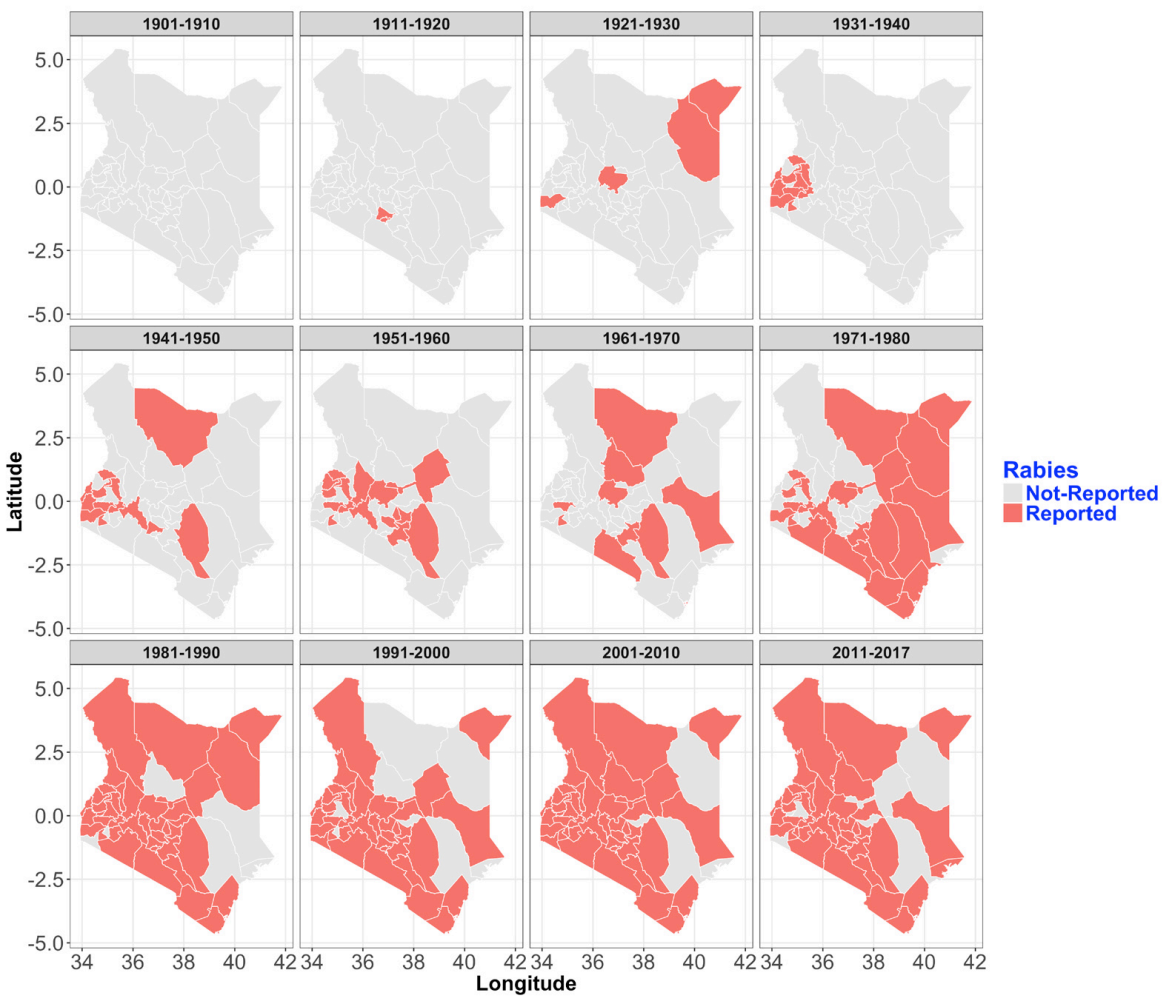
Spatial and temporal occurrence of human and animal rabies in Kenya, 1912 – 2017.

**Figure 5.**
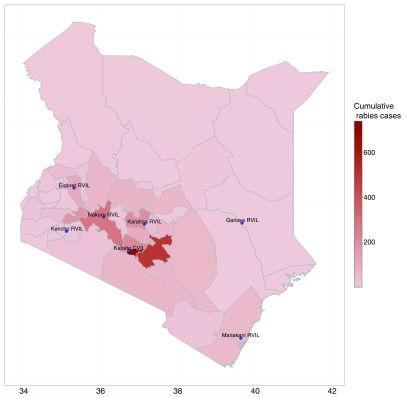
Distribution of confirmed human and animal rabies cases by counties, 1958 – 2017. The blue dots indicate the location of the seven veterinary laboratories (one central Veterinary laboratory and six regional investigative laboratories) in the country. The online version of this figure is interactive.

Historical records show the first case of rabies was in a dog reported in the outskirts of Nairobi in 1912, and the first human case in a woman from the Lake Victoria region in 1928. Our data shows the reported cases were relatively low in numbers and confined to less than 10% of the counties until outbreaks that occurred in the 1940s and 1950s ( Figure 4). Up until 1970, less than half of the counties were reporting rabies cases, and the proportion of samples found positive for rabies was low. The high number of confirmed cases observed in the 1980’s ( Figure 1) was accompanied by increased geographical spread of the disease affecting more than half the counties. Since the 1980’s over 85% of counties in Kenya have consistently reported confirmed cases of rabies ( Figure 4). Cumulatively, 6 of 47 counties (Nairobi, Machakos, Nakuru, Kiambu, Nyeri and Kericho) accounted for nearly two-thirds of all samples submitted and those found positive for rabies ( Figure 5). Each of the six counties has either a veterinary laboratory that carries out FAT or is adjacent to a county with A laboratory with capacity for rabies testing.

## Discussion

Here we have examined data from passive surveillance for rabies in humans and animals in Kenya for the period 1912 – 2017. Although the first official records of the disease date back to 1912, a decade after the establishment of the veterinary department in Kenya, rabies was likely present earlier as local communities in South Nyanza already used the name “swao” to refer to rabies in dogs and jackals ^17^. We were unable to find any literature on the historical emergence of rabies in Kenya, but phylogenetic analysis of rabies viruses in Tanzania show the circulation of two major genetic lineages one of which is thought to have originated from Kenya ^18^.

In the 20 years that followed detection of the first case in Kenya, only sporadic rabies outbreaks were reported in the central and north eastern parts of the country ( Figure 4). By 1950 rabies was present in all counties in the present Western Kenya and was spreading eastwards. The data showed an increase in the number of samples submitted and those confirmed positive upon testing. These increases may be partly explained by changes in surveillance over time (i.e. rates of reporting over time with sample submission), increase in dog population or changes in the incidence of disease associated with rabies control efforts. The regional veterinary investigative laboratories were established within a period of 15 years starting with the laboratory in Nakuru in 1973 followed by Eldoret, Kericho, Garissa, Karatina and Mariakani in 1976, 1979, 1984, 1985 and 1987 respectively. The observed higher burden of rabies in 6 of 47 counties may represent a biased high incidence of rabies associated with close proximity to the regional veterinary investigative laboratories with diagnostic capacity for rabies.

The first systematic attempts to control rabies started with the introduction of a locally produced dog rabies vaccine in the 1950s. Vaccines were delivered through compulsory annual vaccinations, with prosecution of dog owners who did not present their dogs for vaccination. This strategy is reported to have effectively controlled rabies incidence and spread observed until the early 1970s ^8^.

The rapid increase in the number of rabies cases detected and regions affected (from the mid 1970s to the mid 1980s) coincided with the implementation of the Structural Adjustment Policies (SAPs) that privatized veterinary services in Kenya ^19^. The SAPs resulted in marked reduction in the provision of crucial public goods including decreased public funding for veterinary services. The veterinary sector was privatized with government employing fewer veterinary officers and providing reduced public financing for disease control and animal production. The result was a collapse of veterinary services including of the mass dog vaccination campaigns, which may have contributed to the spread of rabies across the country and resulting endemic status.

Data used in this manuscript comes from a passive surveillance system, and are likely a gross underestimate of the total human and animal rabies cases ^1, 20^. Previous studies in East Africa have identified poor surveillance systems and diagnostic capacity leading to underreporting as drivers of the cycle of neglect for rabies ^9, 21, 22^. The incidence of canine rabies estimated from passive surveillance has been estimated to be between one and two orders of magnitude less than estimates from active surveillance in Kenya and Tanzania ^9, 21^.

In the last century, vaccination of dogs has led to the elimination of dog rabies in the U.S.A, Western Europe and elsewhere in the world (see Table 1 in 23) and more recently in some developing countries ^24, 25^. The feasibility of dog rabies elimination in much of Africa is supported by findings that domestic dogs are the reservoirs of the rabies virus and not wildlife, and that most dogs can be reached for parenteral vaccination ^3, 26, 27^.

Although there is evidence rabies that can be eliminated, common misconceptions about rabies epidemiology and transmission among governments in endemic countries may be contributing to the inaction against rabies. These misconceptions that include that rabies is a low priority public health problem, that stray dogs play significant roles in the transmission of rabies, and that wildlife are important reservoir hosts have largely been dispelled through scientific data ^4, 28, 29^. Given the evidence on the feasibility of rabies elimination, and the ranking of rabies as a top priority zoonotic disease in Kenya, the national government developed a 15-year joint human and veterinary sector strategic plan to progressively reduce the burden of rabies in the country with the goal of achieving elimination of dog-mediated human rabies in Kenya by 2030 ^13^.

### Strategic plan for elimination of dog-mediated human rabies in Kenya

**Figure 6.**
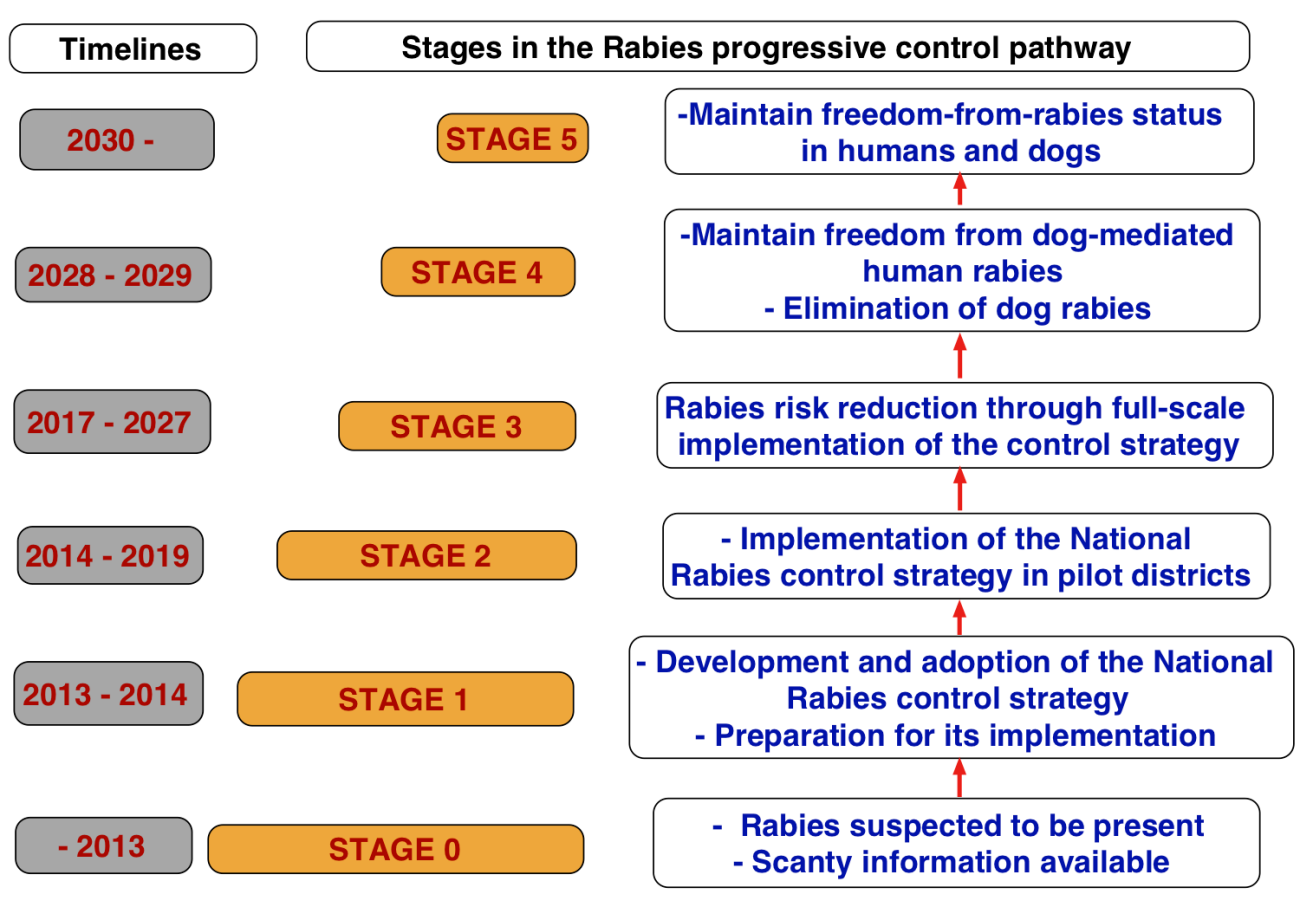
Stepwise Approach to Rabies Elimination (SARE) in Kenya, showing the six stages of the control strategy, associated activities and timelines.

**Figure 7.**
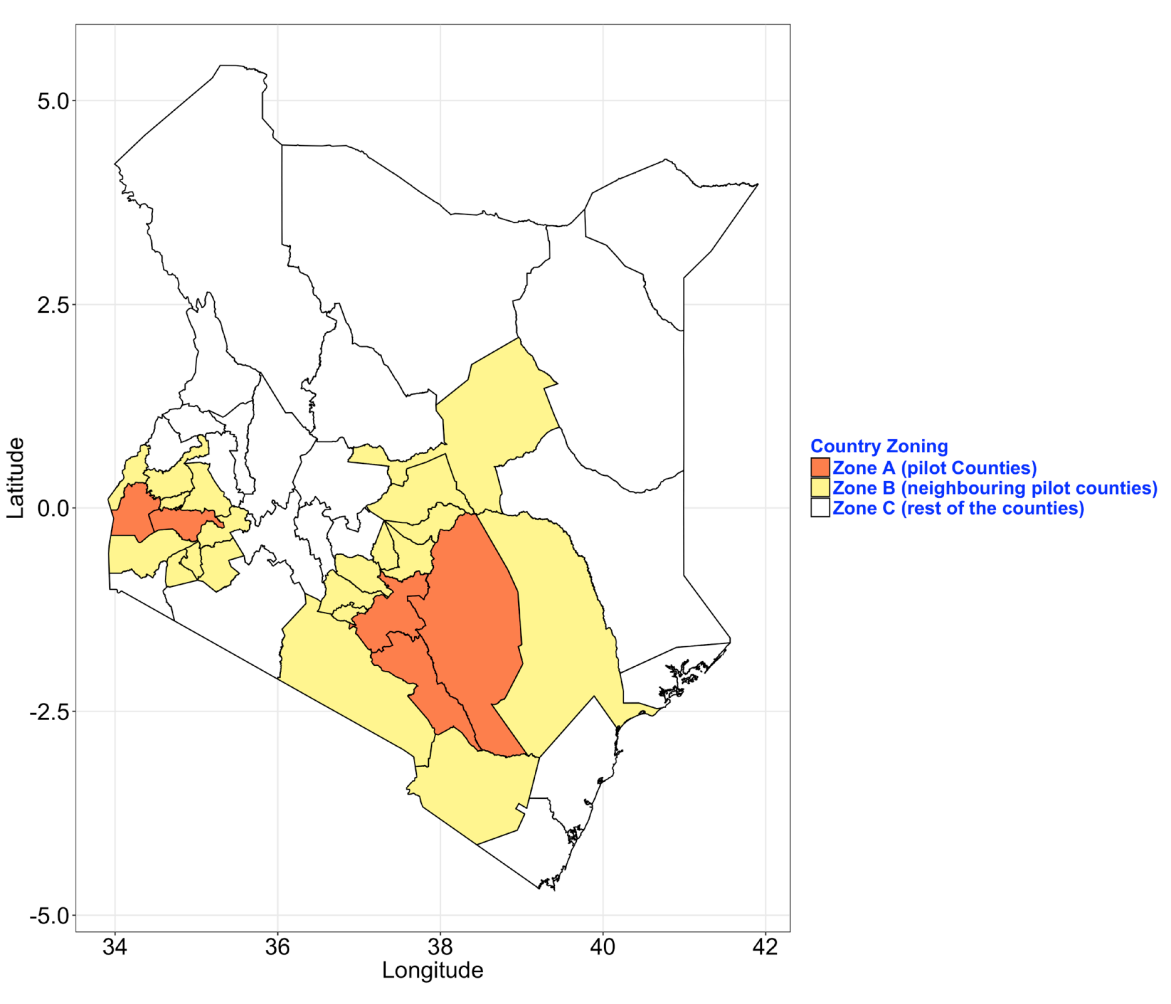
Map of Kenya showing the three zones for the implementation of rabies elimination program. Elimination starts in pilot counties (Zone A), followed by counties neighbouring them (Zone B) and later rolled out in the rest of the counties (Zone C).

The strategy is based on the Stepwise Approach to Rabies Elimination (SARE), a comprehensive risk-based framework that proposes a progressive reduction of disease risk, allowing for coordination of regional activities to achieve disease elimination ^30^. Kenya’s SARE consists of six stages, stage 0 to 5, with a set of activities at each stage building on the previous stage to continuously reduce the risk of disease, until the country is declared free of dog-mediated human rabies at stage 5 ( Figure 6). The initial implementation of the strategy is being carried out in select pilot areas (five counties) to demonstrate success before scaling up to the rest of the country. The pilot areas ( Figure 7, Zone A) were selected to include areas with a high burden of disease (see Figure 5 with the cumulative number of positive rabies samples by county), and areas with and without natural barriers e.g. water bodies and mountains to test the importance of natural barriers in restricting transmission within specific geographical areas. Kisumu and Siaya counties which have natural barriers (Lake Victoria region to the West and Nandi escarpment to the East) and Machakos, Kitui and Makueni Counties which have no defined natural barriers but have reported high numbers of dog and human rabies cases were selected. Experiences and lessons learnt from the pilot regions are being documented and will be used to inform subsequent program scale up to the rest of the country. Scaling up will begin with counties immediately bordering the pilot areas (Zone B) and moving to Zone C ( Figure 7).

The strategy hinges on three main pillars: a) elimination of rabies in dogs through mass dog vaccination, b) prevention of rabies in humans through increased access to post-exposure prophylaxis and public awareness, and c) improved surveillance for rabies in dogs and humans and response to outbreaks.

Domestic dogs are the main source of infection to humans ^3, 6^. The principal method for control of dog rabies is mass vaccination, which has been carried out successfully in many countries including Malaysia, Philippines, Tunisia, and those in Western Europe and North America among others. The OIE and the World Health Organization (WHO) recommends that, to achieve control and eventual elimination of dog rabies, programs must ensure that mass dog vaccination campaigns achieve vaccination coverage of at least 70% of the population in a given area, and that such campaigns are conducted annually for at least three years. This coverage, achieved during a campaign of relatively short duration and using high quality World Organization for Animal Health (OIE) approved dog vaccines that provide long-lasting immunity, is sufficient to maintain the population immunity above the critical threshold for at least 12 months, despite dog population turnover due to births, deaths and migrations during this period without need for a booster dose within the year. This target coverage is supported by validations worldwide investigating the relationship between vaccination coverage and reductions in rabies incidence ^27, 31– 33^.

Evidence from Serengeti ecosystem in Tanzania suggests that domestic dogs are the only population essential for rabies maintenance ^32^. From experiences in Western Europe and North America, rabies elimination in dogs has been successful despite the presence of wildlife hosts capable of transmission where mass dog vaccinations have successfully eliminated the disease from domestic dog populations ^34^. Kenya’s target is to vaccinate at least 70% of dogs in each region annually for at least 3 years to achieve elimination, followed by a maintenance phase with an effective surveillance and outbreak response system. In addition, the national strategy objectives are to provide timely access to post-exposure vaccines for bite patients from suspect rabid dogs, increased public awareness on rabies and establishment of an effective surveillance system for both dog rabies and human rabies.

There is a now growing momentum among countries with endemic dog-mediated rabies to work towards its elimination supported by the Pan Africa Rabies Control Network (PARACON), which assists the coordination of rabies control networks for the different regions in Africa to collaborate and share experiences towards rabies elimination ^30^. Kenya’s strategy is in line with that advocated by the international agencies and builds on a solid body of evidence supporting the 2030 target for countries to have eliminated dog-mediated human rabies ^14^. Data from Kenya has shown that, prior to the Structural Adjustment Program, rabies was relatively better controlled than currently. A functioning veterinary health care system is critical towards achieving rabies elimination. Countries endemic with the disease require to invest in the provision of veterinary services to have effective mass dog vaccination and rabies surveillance systems. Scientific evidence provides strong support that the disease can still be controlled today, and that zero deaths from dog-mediated human rabies by 2030 is a feasible goal for Kenya.

## Data availability

The data underlying this study is available from Open Science Framework http://doi.org/10.17605/OSF.IO/B6WKR ^35^. It contains two datasets - dataset 1: “KenyaRabiesData1958to2017.csv” that produces Figure 1, Figure 2, Figure 3 and Figure 5 and dataset 2: “100YearsRabiesData.csv” that produces Figure 4. The R codes used are provided in a file named “Rcodes_100yearsRabiesinKenya.R”. These datasets are available under a CC0 1.0 Universal license. No records of samples submitted were available for the years 1995, 1996 or 1997.
