# Supplementary material for: A hundred years of rabies in Kenya and the strategy for eliminating dog-mediated rabies by 2030
Source: AAS Open Res. 2019 Feb 15;1:23. Originally published 2018 Jul 30. [Version 2] doi: 10.12688/aasopenres.12872.2 (PMC7117960; doi:10.12688/aasopenres.12872.2)
Supplement: Peer review history

# Peer review: Reviewer response for version 2

**DOI:** 10.21956/aasopenres.14005.r26769

**Published:** 2019-02-25

**Authors:** Tariku Jibat Beyene

**Affiliations:** 1 College of Veterinary Medicine, Center for outcome research and Epidemiology, Kansas State University, Manhattan, KS, USA; 2 College of Veterinary Medicine, Addis Ababa University, Bishoftu, Ethiopia

I thank the authors for a detailed revision of the manuscript. They have addressed my concerns and have considered suggestions/comments. This paper is worth reading for experts working in the field of rabies prevention and control.

I confirm that I have read this submission and believe that I have an appropriate level of expertise to confirm that it is of an acceptable scientific standard.

# Peer review: Reviewer response for version 1

**DOI:** 10.21956/aasopenres.13938.r26556

**Published:** 2018-09-04

**Authors:** Bassirou Bonfoh

**Affiliations:** 1 Centre Suisse de Recherches Scientifiques en Côte d'Ivoire (CSRS), Abidjan, Cote d'Ivoire; 2 Afrique One-ASPIRE, Abidjan, Cote d'Ivoire

This paper is an historical or retrospective study that provide valuable information on the trend of rabies since 1912 and how to eliminate the disease by 2030. The form is good and well structured and provides useful and relevant scientific information. The method is innovative.

 Explain more how the data quality was performed. Were there comments in the annual reports to be considered?  We would like to see the explained link between the structural adjustment of 80s and how that has impacted the outbreak. This will strongly support the proposed strategy. The structural adjustment has dismantled the public services and if we have to reconsider the success of early 70s intervention, we need to compare the current strategy with what has been done before (compulsory vaccination, local vaccine production…). Recommendations should also consider other causes of rabies suspicion (negative samples). The conclusion must comprise the re-organisation of veterinary services as well as the importance of data storage and archives in our countries. This can provide valuable information. We advise the authors to add in their conclusion vet services as key factor for rabies control. The spread of rabies is also due to the neglected aspect of the disease. 

 The comment attached does not alter the content but considered as minors issues to improve the paper

I confirm that I have read this submission and believe that I have an appropriate level of expertise to confirm that it is of an acceptable scientific standard.

# Response

**Published:** 2018-11-27

**Authors:** Thumbi Mwangi

**Affiliations:** Washington State University, Kenya

Many thanks for the very useful review comments and suggestions. We have addressed them and please find below some specific responses.

 Data used in the study came from national records maintained at the central veterinary laboratory and were obtained as a compiled dataset with information on the geographic location and species the samples were obtained from, and the test results. Individual annual reports were not accessed or used. 

 We have provided details (Methods - Data collection – Paragraph 2) of how the data were obtained, reviewed, cleaned and used to create the dataset analysed in this manuscript.

 Paragraph 4 of the discussion section has addressed the structural adjustment programs and their consequence on the provision of veterinary services and disease control including for rabies.

*“The rapid increase in the number of rabies cases detected and regions affected (from the mid 1970s to the mid 1980s) coincided with the implementation of the Structural Adjustment Policies (SAPs) that privatized veterinary services in Kenya ^19^. The SAPs resulted in marked reduction in the provision of crucial public goods including decreased public funding for veterinary services. The veterinary sector was privatized with government employing fewer veterinary officers and providing reduced public financing for disease control and animal production.  The result was a collapse of veterinary services including of the mass dog vaccination campaigns, which may have contributed to the spread of rabies across the country and resulting endemic status.”*

 We have included a statement in the conclusion on the importance of vet services in achieving rabies elimination.

*“A functioning veterinary health care system is critical towards achieving rabies elimination. Countries endemic with the disease require to invest in the adequate provision of veterinary services to have effective mass dog vaccination and rabies surveillance system."*

# Peer review: Reviewer response for version 1

**DOI:** 10.21956/aasopenres.13938.r26554

**Published:** 2018-08-16

**Authors:** Deborah J Briggs

**Affiliations:** 1 College of Veterinary Medicine , Kansas State University, Manhattan, KS, USA

This is an excellent review article detailing the history of rabies across Kenya. The 59 year data set that has been used for the analysis was extracted from laboratory submitted samples in Kenya beginning in 1958 and continuing through 2017. The authors have conducted a thorough analysis of the available data and have explained both the weaknesses and strengths of their analysis. The authors clearly explain some of the contributing factors as to why rabies increased in Kenya in the past and why this deadly disease has been neglected despite having such a high case fatality rate. The authors conclude by detailing the 6 logical steps that Kenya is taking to eliminate rabies throughout the country. Their 15 year strategic plan follows the proven strategies and published recommendations of the WHO, OIE and other international organizations including most importantly, mass vaccination of dogs, improving the access of rabies biologicals for exposed patients, and increasing educational awareness on all levels of society. The paper also details the importance of the final stage of maintenance that includes surveillance on a national level. This paper fills a gap in the history of rabies in Kenya that has been absent until now. It also provides an example for other countries in Africa and elsewhere that are in the fight to prevent human rabies. This paper is a must read for all public health experts working in the field of rabies prevention.

I confirm that I have read this submission and believe that I have an appropriate level of expertise to confirm that it is of an acceptable scientific standard.

# Response

**Published:** 2018-11-27

**Authors:** Thumbi Mwangi

**Affiliations:** Washington State University, Kenya

Many thanks for taking time to review the work we present in this manuscript and for the encouraging comments.

# Peer review: Reviewer response for version 1

**DOI:** 10.21956/aasopenres.13938.r26557

**Published:** 2018-08-14

**Authors:** Darryn L Knobel

**Affiliations:** 1 Center for Conservation Medicine and Ecosystem Health, Ross University School of Veterinary Medicine (RUSVM), Basseterre, Saint Kitts and Nevis; 2 Department of Veterinary Tropical Diseases, Faculty of Veterinary Science, University of Pretoria, Onderstepoort, South Africa

This article reviews historical data on human and animal rabies in Kenya from 1912 to 2017, and gives a broad description of the spatial and temporal trends over this period. The authors also detail the strategy adopted by Kenya towards elimination of dog-mediated human rabies by 2030.

 This is a well-written article that provides a useful synopsis of the available national data on rabies, a notifiable disease in Kenya. While limited by the passive nature of the surveillance system, the data have value in showing historical trends as well as the current status, and the authors are careful to acknowledge the limitations of the data.

 My main comments concern the reproducibility of the results. From what I can make out, the available source data ^1^ is useful to replicate Figure 5 only (Distribution of confirmed rabies cases by counties, 1958-2017). The source data underlying Figures 1-4 do not appear to be available. The authors should make these available or provide a statement if there are issues preventing this.

 More detail, either in the article or as metadata to the source files, is also necessary to describe how the dataset was compiled. For example, I know the administrative units in Kenya have not remained constant over the period of the study. How was this handled in attributing location to specimens?

 There is a lack of detail in the paper regarding the distribution of rabies diagnostic laboratories which should be addressed, given the authors' assertion that this affects surveillance effort and creates bias. The county locations of the CVL and RVIL should be given, to allow the reader to relate this information to Figure 5 and the results presented in the section on spatial distribution of rabies. (For example, the Central Veterinary Laboratory is described as 'in Kabete' but in which county is it [relating to Figure 5]? The second paragraph of the Methods section states that specimens should be submitted to only two other regional laboratories that carry out the FAT [n addition to the CVL], namely Kericho and Mariakani, but in what county are these?) Other regional laboratories are mentioned in the second paragraph of the discussion, implying that these have strengthened the surveillance system, but do these laboratories conduct the FAT? Or are they only mentioned as an indicator of the improvements in the general surveillance system (not specific to rabies)? Why is there no evidence of submission bias around the Mariakani RVIL?

 Although the paper is generally very well written, it would benefit from a further editorial review with a critical eye. For example: the use of a comma separator for 1,000s is inconsistent between the first and second paragraph of the Results; apostrophes should not be used to form plurals of numbers such as "the 1950's" etc. (at least not according to the APA Style); the second and third sentences of the first paragraph of the Discussion should be combined; "US" should be written as "U.S.A." or in full in the 6th paragraph of the Discussion, and the writing in the 7th paragraph of the Discussion needs to be reviewed (e.g. "there is evidence *that* rabies can be eliminated"; ""may be may be contributing"; These misconceptions that include that rabies").

I confirm that I have read this submission and believe that I have an appropriate level of expertise to confirm that it is of an acceptable scientific standard.

## References

[1] : Rabies in Kenya. *Open Science Framework.Web*.2018; 10.17605/OSF.IO/B6WKR

# Response

**Published:** 2018-11-27

**Authors:** Thumbi Mwangi

**Affiliations:** Washington State University, Kenya

*We have provided the two datasets:*

*-  "KenyaRabiesData1958to2017.csv" that is used to produce Figure 1, 2, 3 and 5, and*

*- “100YearsRabiesData.csv” that is used to produce Figure 4.*

*The R codes for the analysis are also provided named “Rcodes_100yearsRabiesInKenya.R”.*

*These datasets and R codes are available under the “Data availability” section of the manuscript through the Open Science Framework -* http://doi.org/10.17605/OSF.IO/B6WKR

 More detail, either in the article or as metadata to the source files, is also necessary to describe how the dataset was compiled. For example, I know the administrative units in Kenya have not remained constant over the period of the study. How was this handled in attributing location to specimens?

*We have included the statement below under the “Data Collection” section to clarify how we attributed location to the specimens submitted.*

*“We obtained records for the years 1912 – 2017 from the Kenya Directorate of Veterinary Services and extracted data on the number of samples submitted for rabies testing, dates of sample submission, animal species and humans, administrative units (counties/districts) where the samples came from, and test results. As the administrative units in Kenya have changed over time, each unit reported in the raw data was matched a current county. The analyses were conducted per existing counties to allow for consistency in reporting.”*

 There is a lack of detail in the paper regarding the distribution of rabies diagnostic laboratories which should be addressed, given the authors' assertion that this affects surveillance effort and creates bias. The county locations of the CVL and RVIL should be given, to allow the reader to relate this information to Figure 5 and the results presented in the section on spatial distribution of rabies. (For example, the Central Veterinary Laboratory is described as 'in Kabete' but in which county is it [relating to Figure 5]? The second paragraph of the Methods section states that specimens should be submitted to only two other regional laboratories that carry out the FAT [n addition to the CVL], namely Kericho and Mariakani, but in what county are these?) Other regional laboratories are mentioned in the second paragraph of the discussion, implying that these have strengthened the surveillance system, but do these laboratories conduct the FAT? Or are they only mentioned as an indicator of the improvements in the general surveillance system (not specific to rabies)? Why is there no evidence of submission bias around the Mariakani RVIL?

*We have included the location of the CVL and RVILs within Figure 5. Under the section “Data collection”, we have clarified on the locations of the central and regional veterinary laboratories and how they operate in regard to rabies diagnosis.*

*“The samples can be sent to the Central Veterinary Laboratory (CVL) located in Nairobi County or to any of six Regional Veterinary Investigation Laboratories (RVIL). The RVILs are across the country in Nakuru, Eldoret, Kericho, Garissa, Nyeri and Kilifi Counties. After initial testing, all samples were further submitted to one of three laboratories: the Central Veterinary Laboratory (CVL), the Regional Veterinary Investigation Laboratories (RVIL) in Kericho in Western Kenya, or RVIL in Kilifi on the Coast. These three laboratories carry out the Fluorescent Antibody Test (FAT), which is the confirmatory rabies diagnostic test recommended by the World Organisation for Animal Health (OIE) ^15^. The test results were reported against the sample’s source species and county of origin.”*

*The Mariakani RVIL is located at the Coast, a region with less cultural practice of keeping dogs associated with the predominant religion and could possibly explain the observation.*

 Although the paper is generally very well written, it would benefit from a further editorial review with a critical eye. For example: the use of a comma separator for 1,000s is inconsistent between the first and second paragraph of the Results; apostrophes should not be used to form plurals of numbers such as "the 1950's" etc. (at least not according to the APA Style); the second and third sentences of the first paragraph of the Discussion should be combined; "US" should be written as "U.S.A." or in full in the 6th paragraph of the Discussion, and the writing in the 7th paragraph of the Discussion needs to be reviewed (e.g. "there is evidence *that* rabies can be eliminated"; ""may be may be contributing"; These misconceptions that include that rabies").

*Many thanks for highlighting these errors – these have been rectified.*

# Peer review: Reviewer response for version 1

**DOI:** 10.21956/aasopenres.13938.r26555

**Published:** 2018-08-07

**Authors:** Tariku Jibat Beyene

**Affiliations:** 1 College of Veterinary Medicine, Center for outcome research and Epidemiology, Kansas State University, Manhattan, KS, USA; 2 College of Veterinary Medicine, Addis Ababa University, Bishoftu, Ethiopia

This study summarizes historical data on rabies in Kenya for 105 yrs and attempts to examine the patterns of rabies spread across the country, and the trends over time. A strategy adopted by Kenya towards the elimination of dog-mediated human rabies by the year 2030 was also discussed.

 My compliments for such a great deal of work in gathering and summarizing a large dataset on rabies in Kenya. 

 Nevertheless, I have the following general and specific suggestion/comments.

**General**

- The study seems to be a mix of a research article (spatiotemporal part) and review (the Kenyan strategy) and lacks a rigorous method for both sections.
- The spatiotemporal part lacks detail on the method of data collection, analysis, and results. For example:
  The objective was to examine the pattern of rabies spread in the country from 1912-2017 but the data in Fig 1-3 shows only for 1958-2017. Thus I advise to clearly state that in the method section.
- The Kenyan strategy aspect of the paper is not mentioned in the methods as well as results section. Was it based on personal opinion or systematic review/ literature search?
- One of the evaluations guidelines of this manuscript asks if the authors provided the necessary data for others to replicate the study. I would recommend the authors to provide the data and R codes used in a usable format. 
- The fact that diagnostic labs were established at different times, comparing the number of samples submitted/tested positive could be tricky as the distance of countries forms labs, public awareness, lab capacities could have influenced it. For instance Figure 2 tells that the percent positive increased over time. Does it mean that the labs used improved methods over time?
- As one of the objectives was to see the trend of rabies overtime, the method section does not explicitly show the method applied. The authors may consider statistical methods to explore the trend. However, a higher number of submitted/reported samples doesn’t mean a high incidence of rabies. Thus, using mixed models where proximity to a lab, the age of the lab (which might have contributed to public awareness) should be included in the analysis to account for the clustering effect.

Specific

- The statement ‘’the first reported case in a dog in the outskirts of Nairobi in 1912, and in a woman from the Lake Victoria basin in 1928’’ was mentioned twice in the MS, one in the second paragraph of the introduction and second, under ‘’spatial distribution of rabies in Kenya’’.
- The last sentence of under the ’spatial distribution of rabies in Kenya’’ could move to discussions section. 
- I think it would be more explicit if table 1 contains info on the region from which the samples came and year. 
- Please make the distinction if numbers given on fig 1-3 are for dogs or all species? As dogs are major sources of rabies for human and others, a line graph or bar graph for all animal spp involved would show the contributions of each over time.
- The third paragraph from the last, discuss ‘’ at least 70% of the population in a given area, and that such campaigns are conducted annually for at least three years. This coverage, achieved during a campaign of relatively short duration, is sufficient to maintain the population immunity above the critical threshold for at least 12 months’’. Could you add how often a booster dose should be provided with a cost-effective way to keep the incidence so low?
- Figure 7, would be good if the names of zones/districts are labeled on the map.

I confirm that I have read this submission and believe that I have an appropriate level of expertise to confirm that it is of an acceptable scientific standard, however I have significant reservations, as outlined above.

# Response

**Published:** 2018-11-27

**Authors:** Thumbi Mwangi

**Affiliations:** Washington State University, Kenya

The study seems to be a mix of a research article (spatiotemporal part) and review (the Kenyan strategy) and lacks a rigorous method for both sections. The spatiotemporal part lacks detail on the method of data collection, analysis, and results. For example: The objective was to examine the pattern of rabies spread in the country from 1912-2017 but the data in Fig 1-3 shows only for 1958-2017. Thus I advise to clearly state that in the method section.

*We have provided a clarification for this correct observation under the data collection section (Methods).*

*For the period prior 1958, data on the number of samples submitted for testing and the species of animals they were obtained from were not available and only aggregate numbers of positive cases of rabies per year per county were available for analyses.*

 The Kenyan strategy aspect of the paper is not mentioned in the methods as well as results section. Was it based on personal opinion or systematic review/ literature search?

*We have included a statement in the methods section on how the strategy was developed and provided a reference linking to the detailed strategy.*

*“This manuscript discusses the strategies put in place for the elimination of human rabies in Kenya by the year 2030. This strategic plan was developed through and elaborate consultative process that involved government departments in health and livestock, research and academic institutions, non-governmental organizations and international partners in Kenya working on public and animal health. The detailed strategy is available online ^13^.”*

 One of the evaluations guidelines of this manuscript asks if the authors provided the necessary data for others to replicate the study. I would recommend the authors to provide the data and R codes used in a usable format. 

*We have provided the datasets and the R codes used for this manuscript under the “Data availability” section*

*“* *The data underlying this study is available from Open Science Framework http://doi.org/10.17605/OSF.IO/B6WKR^35^. It contains two datasets - dataset 1: “KenyaRabiesData1958to2017.csv” that produces Figure 1, 2, 3 and 5 and dataset 2: “100YearsRabiesData.csv” that produces Figure 4. The R codes used are provided in a file named “Rcodes_100yearsRabiesinKenya.R”. These datasets are available under a CC0 1.0 Universal license. No records of samples submitted were available for the years 1995, 1996 or 1997.”*

 The fact that diagnostic labs were established at different times, comparing the number of samples submitted/tested positive could be tricky as the distance of countries forms labs, public awareness, lab capacities could have influenced it. For instance Figure 2 tells that the percent positive increased over time. Does it mean that the labs used improved methods over time? As one of the objectives was to see the trend of rabies overtime, the method section does not explicitly show the method applied. The authors may consider statistical methods to explore the trend. However, a higher number of submitted/reported samples doesn’t mean a high incidence of rabies. Thus, using mixed models where proximity to a lab, the age of the lab (which might have contributed to public awareness) should be included in the analysis to account for the clustering effect.

*We have carried out the analysis using mixed effect models to account for proximity to the testing laboratories. We did not have any data on the changes in the sensitivities of the testing methods or when the exact time when laboratories would have diagnostic methods. This is however unlikely to have caused a major difference since rabies diagnostic methods using brain samples have been known to have be fairly reliable. We tested models with and without the random effect and the results were consistent – an increase in the proportion of samples submitted that were positive for rabies.*

*We have provided details of the data analysis as below – within the methods section.*

*“We determined the proportion of samples submitted that were positive for rabies by year and for each county. Linear mixed-effect models with year as the fixed effect, and the county as a random effect (to account for proximity to testing laboratories) were used to test if the proportion of submitted samples that were positive for rabies changed over time.”*

 The statement ‘’the first reported case in a dog in the outskirts of Nairobi in 1912, and in a woman from the Lake Victoria basin in 1928’’ was mentioned twice in the MS, one in the second paragraph of the introduction and second, under ‘’spatial distribution of rabies in Kenya’’.

*The statement under the “spatial distribution of rabies in Kenya” has been rephrased.*

 The last sentence of under the ’spatial distribution of rabies in Kenya’’ could move to discussions section. 

*We have moved the statement to the discussion section.*

  I think it would be more explicit if table 1 contains info on the region from which the samples came and year. 

*We have 47 regions in the country and including information on region, species and proportion of submitted samples would result in a long difficult to read table. We have however provided all the data as part of the supplementary files (see data availability section).*

 Please make the distinction if numbers given on fig 1-3 are for dogs or all species? As dogs are major sources of rabies for human and others, a line graph or bar graph for all animal spp involved would show the contributions of each over time.

*We have amended the legend of Figures 1 and 2 to indicate these are from all human and animal samples available in our dataset.*
